# Endoscopic thyroidectomy: Our technique

**DOI:** 10.4103/0972-9941.37191

**Published:** 2007

**Authors:** Shailesh P Puntambekar, Reshma J Palep, Anjali M Patil, Neeraj V Rayate, Saurabh N Joshi, Geetanjali A Agarwal, Milind Joshi

**Affiliations:** Galaxy Laparoscopy Institute, 25-A, Karve Road, Near Garware College, Pune - 411 004, India

**Keywords:** Endoscopy, technique, thyroidectomy

## Abstract

Minimally invasive surgery is widely employed for the treatment of thyroid diseases. Several minimal access approaches to the thyroid gland have been described. The commonly performed surgeries have been endoscopic lobectomies. We have performed endoscopic total thyroidectomy by the anterior chest wall approach. In this study, we have described our technique and evaluated the feasibility and efficacy of this procedure.

**Materials and Methods:**

From June 2005 to August 2006, 15 cases of endoscopic thyroidectomy were done at our institute. Five patients were male and 10 were female. Mean age was 45 years. (Range 23 to 71 years). Four patients had multinodular goiter and underwent near-total thyroidectomy; four patients had follicular adenoma and underwent hemithyroidectomy. Out of the seven patients of papillary carcinoma, four were low-risk and so a hemithyroidectomy was performed while three patients in the high risk group underwent total thyroidectomy. A detailed description of the surgical technique is provided.

**Results:**

The mean nodule size was 48 mm (range 20-80 mm) and the mean operating time was 85 min (range 60-120 min). In all cases, the recurrent laryngeal nerve was identified and preserved intact, the superior and inferior parathyroids were also identified in all patients. No patients required conversion to an open cervicotomy. All patients were discharged the day after surgery. All thyroidectomies were completed successfully. No recurrent laryngeal nerve palsies or postoperative tetany occurred. The postoperative course was significantly less painful and all patients were satisfied with the cosmetic results.

**Conclusions:**

It is possible to remove large nodules and perform as well as total thyroidectomies using our endoscopic approach. It is a safe and effective technique in the hands of an appropriately trained surgeon. The patients get a cosmetic benefit without any morbidity.

## INTRODUCTION

Thyroidectomies using the open method are effective, well-tolerated and safe but involve transverse incision on the neck measuring 7–10 cm in length. Thyroid disorders are more common in women and they find these scars uncomfortable and cosmetically unacceptable. Hence, minimal access approaches are playing an ever increasing role in neck surgery as they result in a reduction in size or elimination of the scar on the neck.[1] These approaches to endocrine tumors are more appealing in view of the fact that the conventional approach seems out of proportion compared to the small size of the tumors.[2]

We believe that our endoscopic technique using the anterior chest wall approach as described in this paper can be utilized to remove large nodules of the thyroid as well as performing total thyroidectomies. It combines the benefits of the minimal access approach, instrumentation, magnification and precision. The scars produced are hidden beneath the clothes of the patient offering a cosmetic advantage.

## MATERIALS AND METHODS

Since June 2005, we have performed endoscopic thyroidectomies on 15 patients. The patient demographics are shown in Table 1. Five were men [mean age 45 years (range 27 to 60 years)] and ten were women [mean age 43 years (range 23 to 71 years)]. Four patients had low risk papillary ca and 4 patients had follicular adenomas. Three patients had high risk papillary carcinoma and four had multinodular nodular goitre. The preoperative diagnosis of thyroid tumors was established using fine-needle aspiration cytology and USG neck. Pre and post operative laryngoscopy was done in all patients to confirm vocal cord mobility. The tumor size was < 6 cm in cases of thyroid tumors and the largest dimension of thyroid lobes on USG in multinodular goiters was 11 cm.

**Table 1:** Patient demographics

Age (Years)	Sex	Diagnosis	Nodule size on USG	Surgery
27	m	Papillaryca	4.5 cm	total thyroidectomy
39	f	Papillaryca	3 cm	It hemithyroidectomy with in
60	m	Papillaryca	5 cm	total thyroidectomy
48	f	Follicularad	3 cm	It emithyroidectomy
45	m	Papillaryca	5 cm	total thyroidectomy
33	f	Papillaryca	2 cm	It hemithyroidectomy with in
60	m	Follicularad	6 cm	right hemithyroidectomy
45	m	Mng	7 cm	Subtotal thyroidectomy
71	f	Follicularad	5 cm	It hemi thyroidectomy
25	f	Follicularad	5 cm	right hemi thyroidectomy
35	f	Mng	4 cm	Subtotal thyroidectomy
37	f	Papillaryca	3.5 cm	It hemithyroidectomy with in
23	f	Papillaryca	3.5 cm	It hemithyroidectomy with in
45	f	Mng	7.5 cm	Subtotal thyroidectomy
51	f	Mng	8 cm	Subtotal thyroidectomy

### Surgical technique

The surgical instruments required include one 11 mm trocar and two 5.5 mm trocars, one 10 mm and 5 mm 0 degree fiber optic endoscopes, Harmonic scalpel Ace, 5 mm dissectors, scissors, an aspiration cannula, 5 mm clip applicator and hemostats.

**Figure 1:**
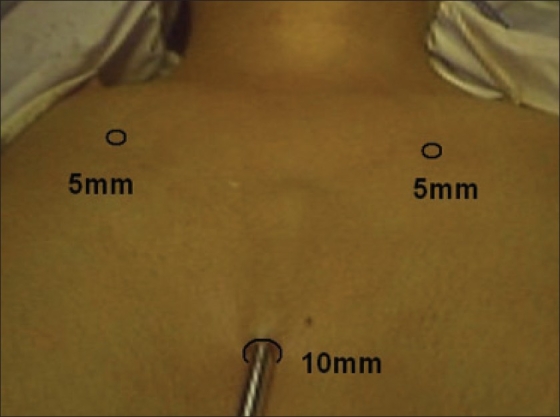
Position of ports

**Figure 2:**
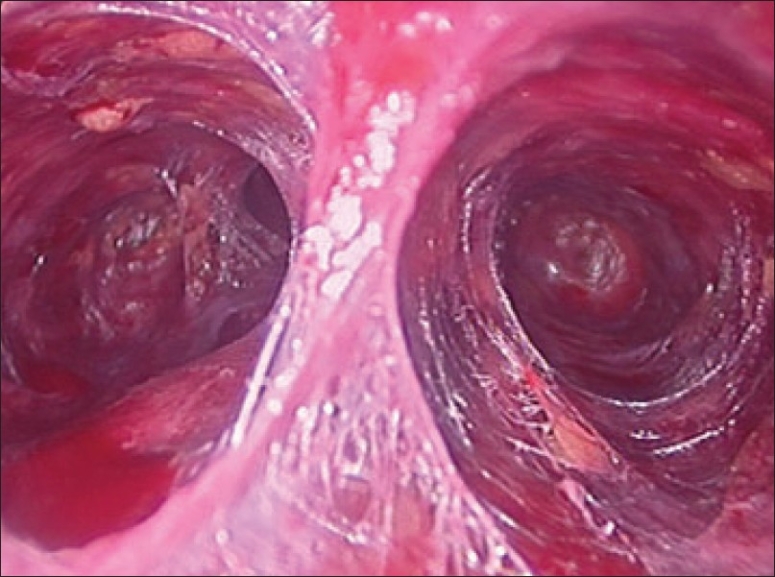
Creating a subplatysmal palne

The procedure was performed with the patient in a supine position under general anesthesia with endotracheal intubation. The neck was extended and the chin was in the midline. A 10 mm skin incision was made on the chest over the sternum about 10 cm from suprasternal notch so as to be covered by the patient's clothes postoperatively [Figure 1]. A long hemostat was inserted through this incision in the subcutaneous plane above the sternum advancing forwards towards the subplatysmal plane as shown in Figure. A 10 mm trocar and cannula was then inserted through this incision. Pneumoinsufflation with carbon dioxide (CO_2_) was begun under endoscopic vision till a continuous pressure of 8 to 10 mmHg was maintained. The gas not only opens up the subplatysmal plane and maintains the operative space, but also may decrease the effect of any minor bleeds [Figure 2].

The subplatysmal space was further developed by blunt dissection with the 10 mm 0 degree rigid endoscope laterally upto the sternomastoid on the left side.

A 5 mm skin incision was made under the clavicle on the left side at the mid-clavicular point. Under endoscopic guidance the first 5 mm trocar and cannula was inserted on the left side and passed over the surface of the clavicle to enter the subplatysmal plane just anterior to the sternocleidomastoid muscle.

The 5 mm Harmonic scalpel Ace was introduced through this port and was used for sharp dissection of the subplatysmal strands, especially in the midline where the platysma is deficient. The subplatysmal plane was further developed upto the hyoid bone superiorly.

The anterior border of the opposite sternocleidomastoid muscle was dissected from the platysma muscle and a space was created laterally.

The second 5 mm trocar was inserted on the right side infraclavicularly at the midclavicular point and a dissector was inserted.

The strap muscles were separated in the midline and retracted laterally to deliver the gland into the operative space.

**Figure 3:**
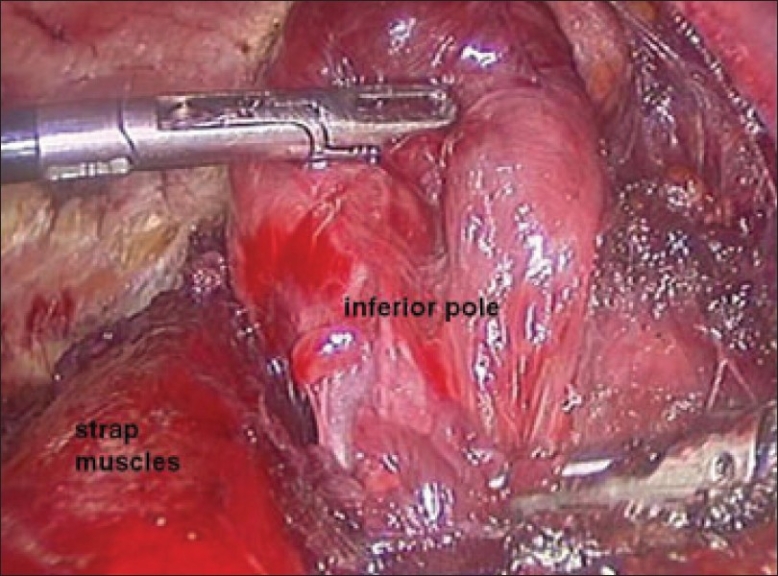
Dissection begins at the inferior pole

**Figure 4:**
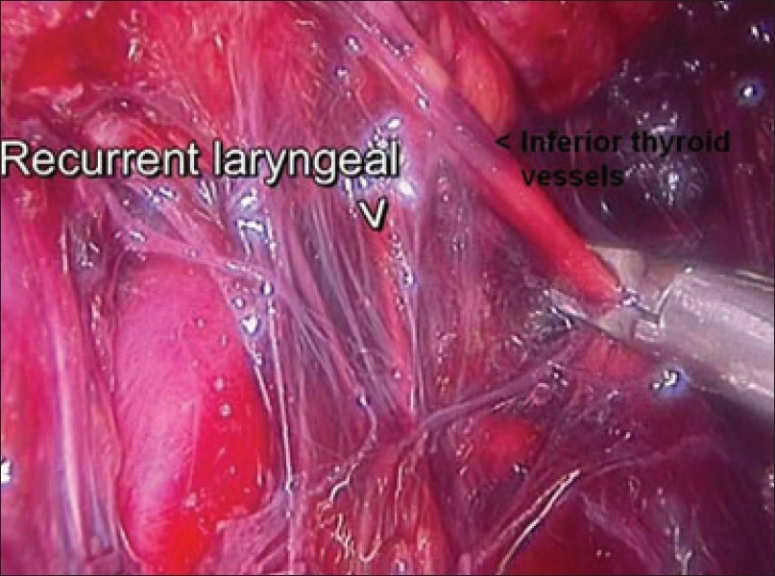
Clipping inferior thyroid vessels. The recurrent laryngeal nerve is identified and preserved

The dissection was begun at the lower pole of the thyroid gland [Figure 3]. The inferior thyroid pedicle was identified. The recurrent laryngeal nerve was also identified and protected. Inferior thyroid veins were first coagulated with the Harmonic scalpel. Inferior thyroid artery was clipped or coagulated with Harmonic scalpel. In doing so, care was taken to avoid injuring the recurrent laryngeal nerve which is usually located between the trachea and the carotid artery [Figure 4].

The inferior parathyroid gland was also identified at this stage.

**Figure 5:**
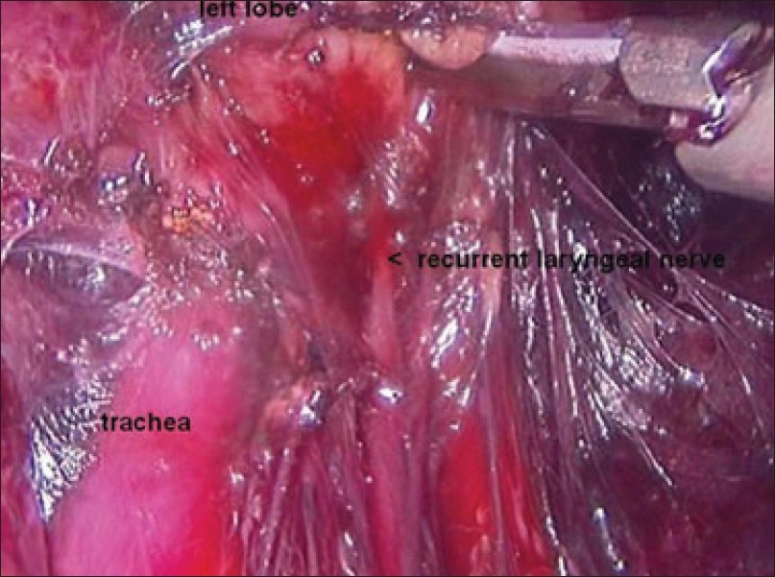
Posterior dissection

**Figure 6:**
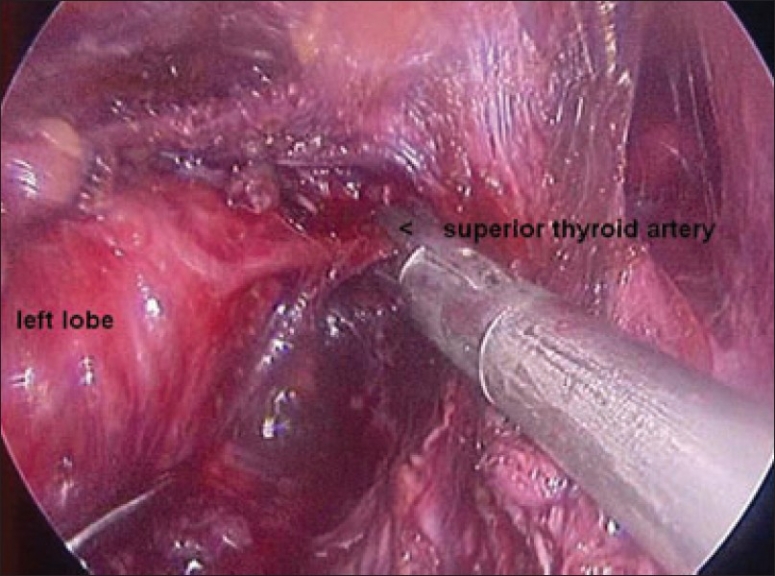
Clipping superior thyroid vessels

Once the inferior pole was freed, the lobe was lifted up from the trachea. A constant traction was maintained on the thyroid lobe medially and the lobe was dissected from the lateral and posterior side keeping the recurrent laryngeal nerve under vision [Figure 5]. The lobe was lifted up from trachea till the superior pole was reached. Then the entire lobe was retracted downwards and the superior thyroid pedicle was taken using clips or coagulated using the Harmonic scalpel [Figure 6]. The superior parathyroid gland was also identified and conserved.

The lobe was retracted laterally and the ligament of Berry was cut with the Harmonic scalpel. The isthmus was also separated from the trachea and cut with the Harmonic scalpel.

The same procedure was repeated on the other side.

**Figure 7:**
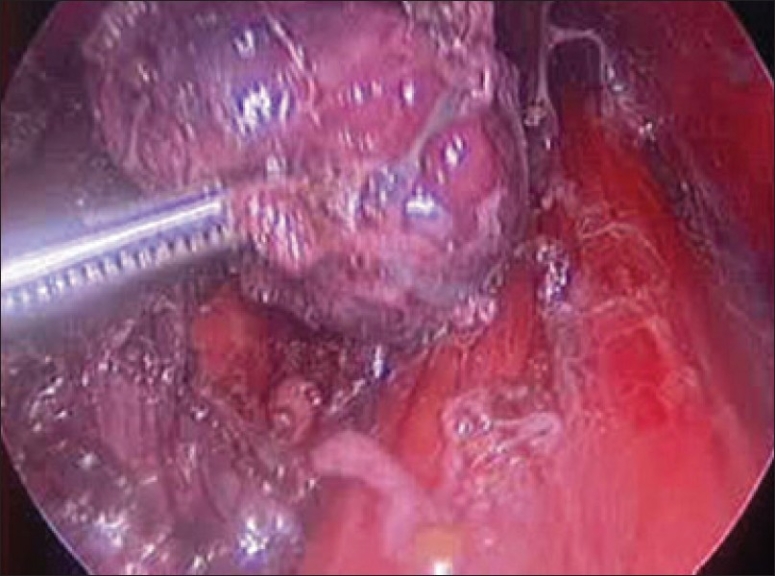
Specimen freed up

The specimen was put in an endobag [Figure 7]. A drain was placed through the 5 mm port.

A 5 mm telescope was put in via the 5 mm port and specimen bag was guided out through 11 mm port. The skin incision on the sternum was widened if required so as to deliver out the specimen.

## RESULTS

Patient demographics and clinical data are shown in Table 1.

Out of the 15 cases, thyroid lobectomy was performed in eight cases, total thyroidectomy in 3 cases, subtotal thyroidectomy in 4 cases. In patients with high-risk papillary carcinoma central compartment lymph nodes were also resected.

The average blood loss was 20 ml (range 15 ml to 35 ml).

Mean operative time was 85 min (range 60 to 120 min).

There were no complications and no cases were converted to open.

There were no cases of recurrent laryngeal nerve palsy or postoperative tetany.

No subcutaneous emphysema, ecchymosis or hypercarbia was observed in any patient.

All patients were discharged on the second postoperative day.

The suprasternal incision was widened to a mean size of 5.6 cm (range 2 to 7.5 cm) for removal of the specimen. However this scar was well hidden beneath the clothes of the patients and all patients were satisfied with the cosmetic result of the surgery.

## DISCUSSION

Videoscopic neck surgery is developing despite the fact that only potential spaces exist in the neck. These approaches are more appealing since the size of incision of that the conventional approach seems to be out of proportion compared to the small size of the tumors.[2]

Gagner first described the endoscopic subtotal parathyroidectomy with constant CO2 gas insufflations for hyperparathyroidism in 1996 and achieved a good clinical and cosmetic result.[3] Since then minimal access parathyroidectomy has found a role alongside conventional cervicotomy for the treatment of primary hyperparathyroidism.

Huscher and colleagues first described the complete endoscopic right thyroid lobectomy in 1997.[4] Minimally invasive surgery using endoscopic vision is now widely employed for the treatment of thyroid diseases for cosmetic purposes. Since then several approaches to the thyroid have evolved including the cervical approach, the minimally invasive video-assisted thyroidectomy (MIVAT), the transaxillary approach and the breast or anterior chest wall approach.[5–9] Each of these approaches have their own advantages and disadvantages.

Endoscopic surgery has reduced the level of surgical “invasiveness” and results in an improved cosmetic appearance. The site of approach is the most important factor because there is an intimate relationship between the locations of the trocars in terms of the cosmetic result, invasiveness, safety and ease of use.[1]

The cervical approach utilizes small incisions in the neck thus making it cosmetically unacceptable and cannot be used for lesions greater than 4 cm. Only patients who have small nodules with a low index of suspected malignancy are offered this endosopic approach.[5] The operative field is small and because the camera is near the anatomic structures, it often has to be removed for cleaning, which significantly increases the operating time.[6]

The axillary approach makes it difficult to visualize the opposite lobe. Although sectioning the sternohyoid muscle creates a good visual space even for the contralateral region and enables the contra lateral gland of the thyroid to be resected, the operating time is extremely prolonged and the additional scar tissue causes discomfort while swallowing and neck pain as a result of adhesions. Therefore this endoscopic procedure is not indicated for thyroid nodules that extend to the contralateral thyroid lobe.[7]

The anterior chest wall approach utilizes port access at various positions on the anterior chest wall depending on the surgeon, thus avoiding a cervical incision. In our technique, the trocars are over the sternum and infraclavicularly. These are hidden by the clothes of the patient and are not visible routinely.[9,10]

This technique also allows bilateral neck exploration. Hence we have been able to perform total thyroidectomies with central compartment clearance for papillary carcinoma and near-total thyroidectomies for large multinodular goiters. The largest dimension of thyroid lobe removed in our series was 11 cm.

The surgeries performed were hemithyroidectomy in eight patients, total thyroidectomy in three patients and near- total thyroidectomies in four patients.

In differentiated thyroid cancer, surgical treatment depends on the risk group of the patient. Low-risk group patients underwent ipsilateral lobectomy and high and intermediate risk group patients underwent near total / total thyroidectomy.

Although the mean nodule size was 48 mm (range 20–80 mm), we have been able to remove lobes upto 11 cm in size in case of multinodular goiter while performing near-total thyroidectomies. These nodules were removed by widening the 10 mm suprasternal incision which is hidden beneath the clothes of the patient. This incision was widened to a mean size of 1.5 cm (range 1.0 to 2.0 cm) for delivery of the specimen. A 5 mm telescope was introduced via the 5 mm port to guide the same under vision.

Our mean operating time was 85 min (range 60-130 min) which is comparable time taken for open cases and the mean blood loss was 20 ml (range 10-50 ml).

Although our number of cases is the same as those reported by Ikeda *et al.*,[10] they have been performed over a period of 15 months while Ikeda *et al.* have reported their experience over 3 years. Our nodule size is larger and our operative time is far less than theirs as is seen in Table 2. Kitano *et al.*[11] have reported a techique of endoscopic thyroid resection using cutaneous elevation in lieu of insufflation by which they have been able to remove large nodules and perform total thyroidectomies with a mean operating time of 270 min. This is the only other reported series in literature of total thyroidectomy.We have performed the same operations in our patients using pneumoinsufflation with CO2 using a pressure of 10 mmHg with a mean operating time of 85 min. By using the lateral approach, Palazzo *et al.*,[12] have removed nodules of mean size 22 mm (range 2–47 mm), while our mean nodule size is 48 mm. The largest dimension of the lobe removed has been 11 cm in our series. Hence the size of the specimen is not a limitation at all, as has been portrayed in the other series. The only other series that reports total thyroidectomies as reported by Miccoli *et al.*,[8] is a multi-institutional experience in which they have used the minimally invasive video assisted approach (MIVAT). They have also reported recurrent nerve palsy in eight cases (2.1%) and eleven patients exhibited hypoparathyroidism, of which only two had permanent hypocalcemia (1.8%). In our series, all cases, the recurrent laryngeal nerve was identified and preserved intact. The superior and inferior parathyroid were identified. Image magnification permits an excellent view of nervous and vascular structures and parathyroid glands.[2] There were no cases of tetany or hoarseness in the postoperative period. Their conversion rate was 4.5% in their series while we in our series no patients required conversion to an open cervicotomy.

**Table 2:** Comparison between reported series of endoscopic thyroidectomy

Series (Approach)	Ikeda (AntChest)	Ikeda (Axillary)	Ikeda (open)	Kitano (skin lift)	Palazzo (Lateral)	Puntambekar (Ant. Chest)
No. of pt.	15	15	15	22	38	15
Av nodule size (mm)	40	42	41	25	22	48
Mean Op time (min)	145	175	84	279	99	85
Av. blood loss (ml)	25	30	36	95	n.a.	20
Av. Hosp stay (days)	4.5	4.2	4.8	4	2	2

The mean blood loss in our series in less than that reported in other series.

All our patients are discharged on the second postoperative day CO2 insufflations at a pressure of 8 mm. Hg is sufficient since only the platysma needs to be lifted. Hence all the complications of gas insufflations i.e., hypercapnia, respiratory acidosis and subcutaneous emphysema are avoided.

Cervical hematomas were not seen in any patients since the Harmonic Scalpel was used which causes denaturation of proteins causing complete hemostasis.[13] Vessels are effectively coagulated without any damage to the surrounding tissue. The 5 mm scissor grip handle connected to the Harmonic Scalpel (Ethicon Endosurgery, Cincinnati, OH, USA), plays an important role in reducing the operating time length.[14]

Surgeons who receive adequate training in endoscopic surgery can perform endoscopic neck surgery as safely as open surgery. We have successfully attempted these cases after a vast experience of performing them by the open method. The chief contraindications to this endoscopic method are previous neck surgery and neck irradiation.[10]

No patient has complained of hyperesthesia or paresthesia in the neck or discomfort while swallowing. All the patients were discharged on the day after surgery and were happy with the cosmetic result.

## CONCLUSION

Endoscopic thyroidectomy via the anterior chest wall approach combines the advantages of minimal access techniques. Inspite the reduced size of skin incision, precise anatomic details are observed through a greatly magnified view using an endoscopic camera. In our series, large nodules have been removed and total thyroidectomy has been done without using cutaneous elevation. Decreased pain and better cosmetic results are the greatest benefits of this procedure.

It also results in decreased functional loss due to transection of the neck musculature after open surgery. Central compartment clearance can be done effectively. Using this technique we postulate the feasibilty of performing modified neck dissections endoscopically.The technique is safe and effective in the hands of an appropriately trained surgeon.
